# Glucocorticoid Effects on the Programming of AT1b Angiotensin Receptor Gene Methylation and Expression in the Rat

**DOI:** 10.1371/journal.pone.0009237

**Published:** 2010-02-16

**Authors:** Irina Bogdarina, Andrea Haase, Simon Langley-Evans, Adrian J. L. Clark

**Affiliations:** 1 Centre for Endocrinology, William Harvey Research Institute, Barts and The London School of Medicine and Dentistry, Queen Mary University of London, London, United Kingdom; 2 Division of Nutritional Sciences, University of Nottingham, Loughborough, Leicestershire, United Kingdom; L' Istituto di Biomedicina ed Immunologia Molecolare, Consiglio Nazionale delle Ricerche, Italy

## Abstract

Adverse events in pregnancy may ‘programme’ offspring for the later development of cardiovascular disease and hypertension. Previously, using a rodent model of programmed hypertension we have demonstrated the role of the renin-angiotensin system in this process. More recently we showed that a maternal low protein diet resulted in undermethylation of the At1b angiotensin receptor promoter and the early overexpression of this gene in the adrenal of offspring. Here, we investigate the hypothesis that maternal glucocorticoid modulates this effect on fetal DNA methylation and gene expression. We investigated whether treatment of rat dams with the 11β-hydroxylase inhibitor metyrapone, could prevent the epigenetic and gene expression changes we observed. Offspring of mothers subjected to a low protein diet in pregnancy showed reduced adrenal *Agtr1b* methylation and increased adrenal gene expression as we observed previously. Treatment of mothers with metyrapone for the first 14 days of pregnancy reversed these changes and prevented the appearance of hypertension in the offspring at 4 weeks of age. As a control for non-specific effects of programmed hypertension we studied offspring of mothers treated with dexamethasone from day 15 of pregnancy and showed that, whilst they had raised blood pressure, they failed to show any evidence of *Agtr1b* methylation or increase in gene expression. We conclude that maternal glucocorticoid in early pregnancy may induce changes in methylation and expression of the Agtr1b gene as these are clearly reversed by an 11 beta-hydroxylase inhibitor. However in later pregnancy a converse effect with dexamethasone could not be demonstrated and this may reflect either an alternative mechanism of this glucocorticoid or a stage-specific influence.

## Introduction

It is recognised that the onset and development of disease in adult life is influenced by the quantity and quality of nutrition during the fetal period [1]–[5]. Epidemiological studies in developed and developing countries have strongly suggested that the intrauterine environment plays a role in determining risk of adult disease. Many cohort studies indicate that lower weight at birth, followed by rapid catch-up growth in childhood, is associated with risk of metabolic syndrome and cardiovascular disease in adulthood [6]–[11]. It has been proposed that maternal undernutrition may “programme” long-term changes in gene expression in the fetus, resulting in cardiovascular and metabolic abnormalities in later life. This epidemiological data is strongly supported by evidence from animal models [12]–[15]. Our laboratory has established a rat model of programmed hypertension and metabolic syndrome, in which the feeding of a low protein diet during pregnancy results in the development of a programmed phenotype in the offspring [16], [17].

There is currently considerable interest in the potential for nutritionally-mediated changes to epigenetic markers in the fetal genome to drive the development of programmed cardiovascular disease. A range of studies using the model of protein restriction during rat pregnancy have suggested that maternal undernutrition leads to reduced methylation and hence increased gene expression of important metabolic and physiological regulators in the offspring [18]–[20]. Heijmans et al (2008) have reported similar hypomethylation of the imprinted IGF2 gene in human subjects exposed to famine during fetal development [21].

We recently demonstrated that maternal protein undernutrition in pregnancy leading to the development of hypertension in the offspring was associated with reduced methylation and increased expression of the At1b angiotensin receptor gene (*Agtr1b*) expression in the rat adrenal gland [22]. Offspring from pregnancies in which the mother had been subjected to an isocaloric low protein diet throughout pregnancy were found to have a number of alterations in expression of components of the renin-angiotensin system in several tissues [23], [24]. The earliest significant changes were found in the expression of the AT1b and AT2 angiotensin receptor in the adrenal [22]. This finding is consistent with increased adrenal responsiveness to angiotensin [25] and observations that the development of hypertension can be prevented by administration of ACE inhibitors or AT1 antagonists in early life [23], [26]. In addition, we were able to show that the low protein diet offspring had reduced methylation of CpG sites in the proximal promoter of the *Agtr1b* implying an epigenetic mechanism may, at least in part, explain this effect [22].

The maternal low protein diet model of programming is a widely used model, but is not unique. Other causes of maternal stress can result in similar long term consequences and this has led to the proposal that stress-related over-activity of the maternal pituitary-adrenal axis in pregnancy results in over-exposure of the fetus to maternal glucocorticoids and that this provides a common mechanism that leads to the changes in gene expression and ultimately the long-term pathological consequences associated with programming. In support of this hypothesis, administration of dexamethasone in pregnancy also has similar long term cardiovascular consequences [27], [28].

In this study we wished to test the glucocorticoid hypothesis using *Agtr1b* expression and epigenetic changes as molecular end points. The most direct test of the hypothesis is to expose pregnant animals receiving a low protein diet to the 11 β-hydroxylase inhibitor, metyrapone which blocks the last steps of corticosterone synthesis in the rat [29]. We have previously shown that this protocol normalizes the blood pressure of the resulting offspring of protein restricted rats, strongly suggesting that steroids play a key role in mediating the programming effects of undernutrition [24], [27]. If glucocorticoids are central to the low protein effects, metyrapone should block the early change in gene expression. As in our previous reports this study targeted the blockade to the first 14 days of gestation, as prolonged use of this drug in pregnancy can inhibit lung maturation in the fetus. For comparison with another model of programmed hypertension we studied offspring of mothers treated with dexamethasone during pregnancy. In this second experiment, the final week of gestation was targeted for intervention as this has been previously shown to be the period of maximal sensitivity of rat fetal development to glucocorticoid exposure [27], [30]. In all cases offspring were studied postnatally for alterations in *Agtr1b* expression and DNA methylation at 1 week of life and at 4 weeks for blood pressure.

## Results

### Metyrapone Study

**Figure 1:**
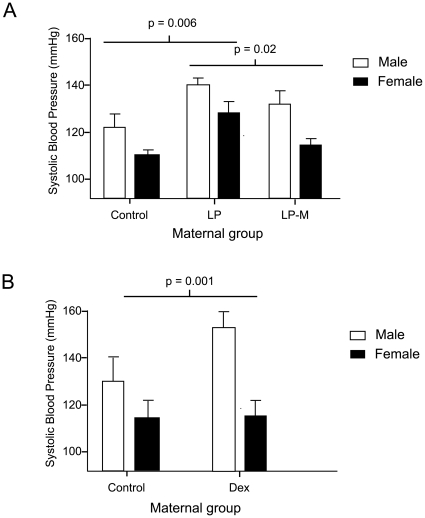
Blood pressure at 4 weeks of age in offspring. A) rats fed low protein diet throughout pregnancy without (LP) or with metyrapone over days 0–14 gestation (LPM), and B) rats treated with dexamethasone over days 15–22 gestation. Data are shown as mean ± SEM for 8 observations per group. Two way ANOVA indicated that in the metyrapone experiment blood pressure was influenced by maternal treatment (p = 0.006), but there was no effect of sex. In the dexamethasone study, blood pressure was influenced by dexamethasone treatment (P<0.001) and by the interaction of sex and treatment (p = 0.049). All measurements were corrected for body weight.

8 litters from mothers fed (a) a control diet, (b) a low protein diet or (c) a low protein diet with metyrapone administered for the first fourteen days of pregnancy were studied. Consistent with our previous reports [16], [24] neither low protein feeding, nor treatment with metyrapone had any significant impact upon maternal weight gain in pregnancy, reproductive outcome or birth weights of the offspring (**Table 1**). Offspring were weaned onto standard laboratory chow and blood pressure was measured at four weeks of age. Blood pressure is unlikely to be altered at earlier time points and is technically more difficult to measure. **Figure 1a** shows that both males and females subjected to low protein during fetal life had significantly greater systolic blood pressure, whereas animals whose mothers were treated with metyrapone maintained control levels of blood pressure. A 2 way ANOVA for sex and maternal treatment was performed and this showed no effect of sex (F = 1.737, p = 0.146), an effect of treatment (F = 4.837, p = 0.013) and no interaction of sex and treatment (F = 0.375, p = 0.689).

**Table 1:** Maternal weight gain, food intake and reproductive outcomes following protein restriction and metyrapone administration over d0–14 gestation.

Maternal diet Treatment	Control Saline	LP Saline	LP Metyrapone
Weight at mating (g)	247±7	253±6	244±7
Weight at delivery (g)	383±12	402±8	387±8
Food intake d0–7 (g/day)	29.2±2.6	32.4±1.4	30.6±0.9
Food intake d8–14 (g/day)	26.4±2.0	27.7±1.2	26.6±1.9
Food intake d15–22 (g/day)	26.5±2.8	23.5±0.7	26.0±2.0
Litter size (no. pups)	12±1	13±1	14±1
Birth weight male offspring (g)	5.60±0.12	5.23±0.16	5.61±0.25
Birth weight female offspring (g)	5.18±0.14	4.96±0.23	5.40±0.19

Data are shown as mean ± SEM for 8 observations per group. There were no significant differences between the groups.

**Figure 2:**
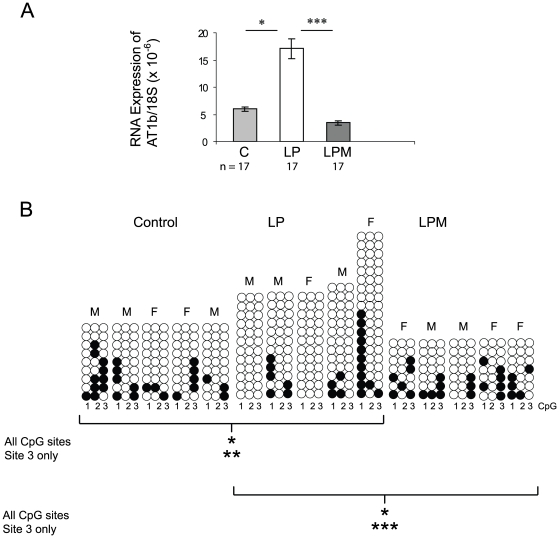
Effect of Metyrapone on *Agtr1b* expression and DNA methylation. (A) Gene expression in animals at 1 week of age whose mothers received control (C) or low protein diets during pregnancy with vehicle alone (LP) or metyrapone (LPM) for the first fourteen days of pregnancy. Results are expressed relative to the 18S ribosomal RNA and are expressed as the mean ± SEM. The number of adrenals studied in each group is shown below each column. * p<0.05; *** p<0.005 (B) Methylation of the three most proximal CpG sites in the *Agtr1b* promoter in the adrenal at 1 week of age. Each empty circle represents an unmethylated CpG site, while filled circles represent methylated sites. Each column represents a single adrenal, the sex of which is shown above the column. Each horizontal group of three circles indicates a single sequenced clone. Differences between groups are calculated by Chi squared. * p<0.05; ** p<0.01; *** p<0.005.

Adrenal glands were harvested from 1 week old animals and used to make cDNA and genomic DNA. This time point was chosen as previous work had shown that RNA expression changes were statistically significant at this time point (22), whereas at later time points changes in expression of some genes were likely to be secondary to development of hypertension. If changes in gene expression at 1 week were related to DNA methylation we would expect to identify such methylation changes in adrenals at this age also. Real-time PCR was performed on these samples using *Agtr1b* and 18S RNA primers. As we have shown previously, *Agtr1b* mRNA expression was significantly greater in MLP adrenal offspring, but administration of metyrapone completely prevented this overexpression (**Figure 2a**).

Genomic DNA prepared from these adrenals at 1 week of age was bisulphite converted, amplified and subcloned. Individual clones from adrenals from 5 different animals in each treatment group were sequenced to determine the degree of methylation of the three CpG sites in the AT1b proximal promoter. As we reported previously, the MLP offspring had less methylation at these sites compared to controls (controls, 20.0%; LP, 10.8%; p<0.05). Administration of metyrapone in early pregnancy significantly reversed this trend though, such that methylation levels were indistinguishable from controls and significantly greater than the LP group (LP, 10.8%; LPM, 23.8%; p<0.05). These changes were most clearly reflected in the methylation status of CpG site 3 (control, 28.9%; LP, 5.4%; LPM, 40%) (**Figure 2b**).

### Dexamethasone Study

**Figure 3:**
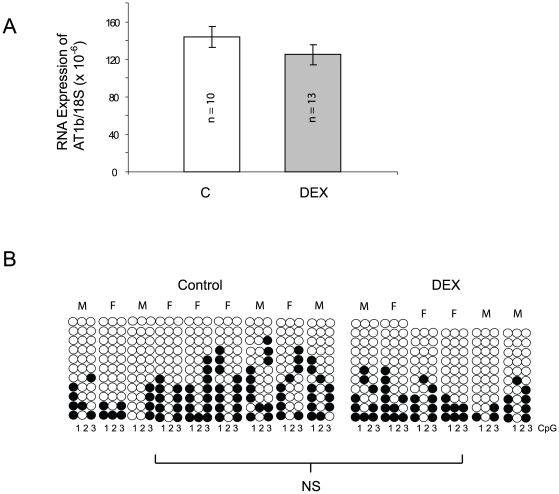
Effect of Dexamethasone on *Agtr1b* expression and DNA methylation. (A) Expression of the *Agtr1b* at one week of age in animals whose mothers received control (C) or dexamethasone during the last 7 days of pregnancy. Results are expressed relative to the 18S ribosomal RNA and are expressed as the mean + SEM. The number of adrenals in each group are shown on the columns. There were no statistically significant differences. (B) Methylation of the three most proximal CpG sites in the *Agtr1b* promoter in the adrenal at 1 week of age. Each empty circle represents an unmethylated CpG site, while filled circles represent methylated sites. Each column represents a single adrenal, the sex of which is shown above the column. Each horizontal group of three circles indicates a single sequenced clone. The differences between the two groups were not statistically significant.

For comparison with a distinct model of hypertension programmed *in utero*, rats were administered dexamethasone during pregnancy. **Table 2** shows the maternal response to dexamethasone administration, which had no significant impact upon either gestational weight gain or reproductive outcomes. Surprisingly, at this administered dose of dexamethasone there was no suppression of food intake and in fact the pair-fed controls had a non-significant trend towards slightly less food consumption than the treatment group over the final week of gestation. Pups born to dexamethasone treated dams were of normal birth weight. At four weeks of age a similar effect on blood pressure to that observed after the MLP diet was observed and blood pressure was influenced by dexamethasone treatment (P<0.001) and by the interaction of sex and treatment (p = 0.049) (**Figure 1b**).However no difference in adrenal *Agtr1b* expression at one week of age could be identified (**Figure 3a**). Furthermore, analysis of the same proximal promoter CpG sites from 9 controls and 6 treated adrenals showed very similar degrees of methylation (Control 26.9%; Dex treated 24.2%; not significant) (**Figure 3b**).

**Table 2:** Maternal weight gain, food intake and reproductive outcomes following dexamethasone administration over d15–22 gestation.

	Control (pair fed)	Dexamethasone treated
Weight at mating (g)	268±6	262±7
Weight at delivery (g)	416±13	417±7
Food intake d0–7 (g/day)	25.6±0.6	26.3±0.7
Food intake d8–14 (g/day)	29.1±0.5	29.8±0.6
Food intake d15–22 (g/day)	29.3±0.9	29.8±0.7
Litter size (no. pups)	14±1	15±1
Birth weight male offspring (g)	6.67±0.24	6.65±0.17
Birth weight female offspring (g)	6.42±0.20	5.88±0.25

Data are shown as mean ± SEM for 8 observations per group. There were no significant differences between the two groups.

## Discussion

In this study we have reproduced the findings reported in our previous paper showing that at one week of postnatal life, rat offspring that were exposed to a maternal low protein diet *in utero* have increased expression of the adrenal *Agtr1b* and reduced methylation of CpG residues in its proximal promoter [22]. By the age of four weeks these animals already showed evidence of hypertension. The novel finding we report here however is that when the mothers receive metyrapone during the first two weeks of pregnancy the hypertension, the increased *Agtr1b* expression and the reduced DNA methylation were all normalised. The findings of this study are, therefore, highly suggestive of the maternal pituitary-adrenal axis playing a central role in mediating the adverse effects of the low protein diet and this would be consistent with widely held views [31].

Alternative explanations should be considered however. One possibility is that metyrapone has induced a long-term effect on adrenal development and differentiation such that 1 week old animals are hypoadrenal and consequently protected from developing hypertension. Though unlikely in view of the extensive experience in the use of this drug, it is notable that the size of adrenals did not differ in the metyrapone group (data not shown). In the rat fetus, the adrenal exists as a distinct organ at 14 days, but has no evidence of zonation. The *Agtr1b* is not expressed at this early stage [32], [33]. Consequently this data suggests that a maternal glucocorticoid effect is able to ‘programme’ the expression of the *Agtr1b* at an early, pre-zonation, stage of adrenal development.

One explanation for these observations is that activated glucocorticoid receptor binds to the two functional GREs in the *Agtr1b* [34], [35] and that this process consequently impairs the laying down of DNA methylation that would normally take place during this phase of differentiation and development. A large number of genes contain GREs and this might be a very detrimental process if it applied to all glucocorticoid responsive genes in the fetus. Therefore it seems probable that there is greater complexity and specificity in effecting this response. Alternatively, the DNA region around CpG site 3 in the *Agtr1b* forms a perfect *polycomb* response element. Such elements are complex and include binding sites for several proteins [36] and may act as transcriptional repressors or activators of particular importance in early development [37]–[40]. Precisely how the DNA methylation observed at this site would relate to *polycomb* activity which would normally result in specific histone methylation marks is not clear at present.

Although confirmatory of earlier studies [24], [41], the finding that dexamethasone in the final week of gestation could elevate blood pressure in the offspring independently of any effects upon maternal food intake is important. Woods and colleagues have asserted that rather than glucocorticoids mediating programming effects of undernutrition, all effects of steroid treatment in rodent pregnancy are related to associated reductions in food intake [31]. Our findings refute this assertion. The selectivity of the effect is demonstrated by our observation that dexamethasone administration does not lead to increased *Agtr1b* expression or alteration of methylation. Interestingly we also noted a strong sex-specific effect in the dexamethasone study consistent with our previous findings [24].

The distinct characteristics of the two hypertension-inducing treatments used in this study may reflect the different stages in pregnancy at which each of the drugs was administered. It could be inferred that that early glucocorticoid exposure results in *Agtr1b* changes, whereas later exposure acts upon blood pressure regulation through an independent, sexually dimorphic mechanism. Experiments with prenatal protein restriction indicate that the insult can induce hypertension at any stage of pregnancy [16], [42], but that effects are greatest if the undernutition is induced later in gestation. Alternatively, it could be argued that dexamethasone, in contrast to corticosterone, will not be susceptible to conversion into inactive steroid by 11 β-hydroxysteroid dehydrogenase in the placenta and will almost certainly provide a more potent glucocorticoid stimulus to the developing adrenal than maternal corticosterone could do. Dexamethasone lacks significant mineralocorticoid activity [43], whereas endogenous maternal corticosterone has a potent mineralocorticoid effect in addition to maternal aldosterone. Treatment of animals with metyrapone inhibits both corticosterone *and* aldosterone synthesis, perhaps suggesting that mineralocorticoid receptor dependent mechanisms are important in programming AT1b gene expression at this earlier stage of pregnancy.

In conclusion, these studies confirm our earlier observations linking the adverse effects of fetal programming events to epigenetic mechanisms and strongly suggest a role for maternal glucocorticoid in this process. Epigenetic mechanisms have been associated with the consequences of programming in several models affecting for example the glucocorticoid receptor, PPARα and DNA methyltransferase [18]–[20]. Such observations suggest a highly plausible mechanism leading to long-term adverse consequences. The major challenges now seem to be in understanding how the prenatal insult brings about the epigenetic changes observed and in demonstrating the causative, as opposed to reactive role for such changes.

## Materials and Methods

### Animals

The experiments described in this report were performed under license from the Home Office in accordance with the 1986 Animals (Scientific Procedures) Act. The study used rats of the Wistar strain, and all animals were housed in plastic cages and subjected to a 12 hour light/dark cycle at a temperature of 20–22°C.

#### Metyrapone study

Twenty-four virgin female Wistar rats (Harlan Ltd, Belton, Leics, UK) were mated at weights between 250 and 275 g. Upon confirmation of mating by the appearance of a semen plug on the cage floor, the rats were allocated to be fed (*ad libitum*) isoenergetic diets containing 18% protein (control, n = 8) or 9% protein (LP, n = 16), as described previously [27]. Half of the LP fed rats received twice daily injections of metyrapone (5 mg/kg body weight, in saline s.c group LPM) over days 1–14 of pregnancy. Control rats and the remaining LP rats received vehicle injections. Metyrapone was administered at a dose previously shown to have no adverse effect upon reproductive outcome and to reduce maternal corticosterone concentrations by 90% [29], [44]. In unpublished work we have found that higher doses led to fetal abnormalities. At delivery the litters were culled to a maximum of eight pups (4 males and 4 females) to minimize variation in suckling nutrition. Offspring were culled using CO_2_ asphyxia and cervical dislocation at 1 or 4 weeks of age (1 male and 1 female per litter at each time point).

#### Dexamethasone study

Sixteen virgin female Wistar rats (250–275g) were fed standard laboratory chow diet and were mated with a stud male. At day 15 gestation the rats were allocated for either vehicle injection (n = 8, 0.1 ml saline, s.c. daily) or treatment with dexamethasone (n = 8, 100µg/kg body weight daily, s.c. injection). As dexamethasone has been reported to reduce food intake in rats, all vehicle-treated, control animals were pair-fed to the dexamethasone group. Treatment continued until the females gave birth on day 22 of gestation. At delivery the litters were culled to a maximum of eight pups (4 males and 4 females). Offspring were culled at 1 or 4 weeks of age (1 male and 1 female per litter) as previously. These timepoints were selected as it is established that elevated blood pressure is in place by 4 weeks [16] and the earlier timepoint enabled evaluation of any adrenal changes ahead of any influences of hypertension.

### Blood Pressure Determination

Systolic blood pressure was determined using an IITC Model 229 Blood Pressure monitor as reported previously [16], [23], [25]. This tail-cuff method has been extensively validated and refined to reduce possible stress-related effects and observer subjectivity. Rats were placed in a darkened room maintained at 27°C for 2 h and settled in a Perspex tube. A suitably sized cuff was placed over the tail and inflated to 300 mmHg. Pulses were recorded during deflation at a rate of 3 mmHg/s. Blood pressure, which was determined in triplicate for each animal, was derived using a preset algorithm via appropriate software. The average systolic pressure from the three measures was recorded. We have previously demonstrated that we can acquire reproducible results without the need for training of the animals to the procedure [23]. It has been demonstrated that values obtained using this method are similar to those obtained under direct anaesthesia [45]. Our earlier observations of raised tail-cuff pressures in animals subject to prenatal protein restriction have been replicated in studies using both radiotelemetry and swivel-cannulation systems [46], [47], as have studies which have focused upon antenatal glucocorticoid administration to induce hypertension [48].Data analysis included correction for body weight.

### RNA Quantitation

Some of the offspring were culled at 7 days of age and total RNA was isolated with Agencourt RNAadvance Tissue total RNA purification kit (Agencourt, Beverley, MA, USA), which uses paramagnetic bead-based technology in a 96-well format. Frozen adrenals (2–10 mg) were homogenized (Precellys 24 benchtop equipment, Montigny-le-Bretonneux, France). Half of the sample was frozen for further DNA isolation and the remainder used for RNA isolation. RNA was immobilized onto the paramagnetic particles, treated with DNaseI (RNase-free, Ambion, Warrington, UK)) and washed. Nucleic acid concentration was measured by a Nanodrop ND1000 spectrophotometer.

cDNA was synthesized using MMLV reverse transcriptase (Promega, Southampton, UK).

QPCR was performed using a 2-step cycling protocol: 95°C×10 minutes, then 40 cycles of 95°C×30 s and 59°C×1 minute on the MX4000 (Stratagene, La Jolla, CA, USA). Primers and probe were as described [22]. Amplification plots were analyzed using MX4000 software version 3.0 (Stratagene). RNA expression data were given as copy number of gene of interest/18S RNA. Standards used were PCR fragments purified from a polyacrylamide gel. All PCR reactions were performed in triplicate.

### Methylation Detection

Genomic DNA was isolated using the QIAamp DNA mini kit (Qiagen, Crawley, UK). 0.5 µg DNA was subjected to bisulphite treatment using EZ DNA Methylation-Gold Kit ™ (ZYMO Research, Orange, CA, USA) according to manufacturer's instructions. 4µl of DNA was used in PCR with primers designed to the reverse strand bisulphite-converted DNA [22]. PCR conditions were: 95°C×10 minutes, then 40 cycles of 95°C×30 s, 52°C×1 minute, 72°C×30 s and finally 1 cycle of 7 minutes ×72°C. The reaction mixture contained 1x PCR Gold buffer, 0.2 mmol/L dNTPs, 2 mmol/L MgCl_2_, 1 µmol/L primers and 1.25U of Ampli*Taq* Gold DNA polymerase (Applied Biosystems). Gel purified PCR products were passed through SNAP columns (Invitrogen, Paisley, UK), cloned into pCR4- TOPO vector (Invitrogen) and sequenced.

### Statistical Analysis

All data was analysed using the Statistical Package for Social Sciences (SPSS, Inc, Chicago, IL, Version 14.0). Differences between groups were assessed using a mixed model ANOVA (fixed factors, maternal treatment, age and offspring sex), unless otherwise indicated in the text. A Bonferroni test was applied to correct for multiple testing. Values are expressed as mean ± S.E.M. P<0.05 was considered as significant. As multiple pups from the same dam were used throughout this study, litter of origin was included as a fixed nested factor in all analyses. The frequency of cytosine methylation was compared using chi squared analysis_._
